# The Outcome of Antibiotic Overuse before and during the COVID-19 Pandemic in a Tertiary Care Hospital in Oman

**DOI:** 10.3390/antibiotics12121665

**Published:** 2023-11-27

**Authors:** Nenad Pandak, Hilal Al Sidairi, Ibrahim Al-Zakwani, Zakariya Al Balushi, Shabnam Chhetri, Muna Ba’Omar, Sultan Al Lawati, Seif S. Al-Abri, Faryal Khamis

**Affiliations:** 1 Department of Infectious Diseases, Royal Hospital, P.O. Box 1331, Muscat 111, Oman; zak077@gmail.com (Z.A.B.); drshabnamchhetri24@gmail.com (S.C.); muna.baomar@gmail.com (M.B.); smhlawati@gmail.com (S.A.L.); salabri@gmail.com (S.S.A.-A.); khami001@gmail.com (F.K.); 2 Department of Microbiology, Royal Hospital, P.O. Box 1331, Muscat 111, Oman; hilal.alsidairi@moh.gov.om; 3 Department of Pharmacology and Clinical Pharmacy, College of Medicine and Health Sciences, Sultan Qaboos University, P.O. Box 1331, Muscat 111, Oman; azakwani@squ.edu.om

**Keywords:** antibiotics, multidrug resistance, meropenem, vancomycin, ceftriaxone, pandemics, COVID-19, *Candida auris*, Oman, viral infections

## Abstract

Antimicrobial resistance (AMR), a serious global public health challenge, may have accelerated development during the COVID-19 pandemic because antibiotics were prescribed for COVID-19. This study aimed to assess antibiotics use before and during the pandemic and correlate the results with the rate of resistant microorganisms detected in hospitalized patients during the study period. This single-center study looked retrospectively at four years of data (2018–2021) from Royal Hospital, Muscat, which is the biggest hospital in Oman with approximately 60,000 hospital admissions yearly. The consumption rate of ceftriaxone, piperacillin tazobactam, meropenem, and vancomycin was presented as the antibiotic consumption index, the ratio of defined daily dose (DDD) per 100 bed days. Analyses were performed using the nonparametric test for trend across the study period. Correlation between antibiotic consumption indexes and the isolated microorganisms in the four-year study period was performed using Spearman’s rank correlation coefficient. We compared data from the pre-COVID-19 to the COVID-19 period. Though more patients were admitted pre-COVID-19 (132,828 versus 119,191 during COVID-19), more antibiotics were consumed during the pandemic (7350 versus 7915); vancomycin and ceftriaxone had higher consumption during than before the pandemic (*p*-values 0.001 and 0.036, respectively). Vancomycin-resistant Enterococcus (VRE) and *Candida auris* were detected more during the COVID-19 period with *p*-values of 0.026 and 0.004, respectively. Carbapenem-resistant Enterobacterales (CRE), vancomycin-resistant *Enterococcus* spp., and *C. auris* were detected more often during the pandemic with *p*-values of 0.011, 0.002, and 0.03, respectively. Significant positive correlations between antibiotic consumption and drug-resistant isolates were noted. This study confirms that the overuse of antibiotics triggers the development of bacterial resistance; our results emphasize the importance of antibiotic control.

## 1. Introduction

Antimicrobial resistance (AMR) presents a serious challenge to public health globally. There were an estimated 4.95 million deaths associated with bacterial AMR in 2019, including 1.27 million (95% UI 0.911–1.71) deaths attributable to bacterial AMR [1], and it has been assessed that this will increase to 10 million deaths annually by the year 2050 unless appropriate actions are taken [2]. AMR naturally occurs as a mechanism by which microorganisms adapt to survive in hostile environments like those containing antibiotics. There are multiple drivers of AMR such as lack of access to clean water, sanitation, and hygiene for both humans and animals; poor infection prevention and disease control in health care facilities and farms; poor access to quality, affordable medicines, vaccines, and diagnostics; lack of awareness and knowledge; and lack of enforcement of legislation. However, the inappropriate use (misuse and/or overuse) of antibiotics seems to be the major contributing factor in the emergence of AMR [3]. 

It has been almost four years since the world witnessed the onset of COVID-19 and its newly identified causal agent, SARS-CoV-2. Recommended treatment options for COVID-19 include antiviral drugs, corticosteroids, immunomodulatory drugs, and low molecular weight heparin, as well as antibiotics [4]. Although COVID-19 is a viral disease and antibiotics are indicated for bacterial, not viral, infections, many studies demonstrate that antibiotics are prescribed regardless to treat COVID-19 [5,6]. 

The rationale for antibiotic COVID-19 treatment was based on three different reasons. The first was not only the previous experience with influenza caused by different influenza viruses, but the experience with other coronavirus respiratory diseases such as SARS (severe acute respiratory syndrome) and MERS (Middle East respiratory syndrome). In these viral pneumonias, a bacterial infection was present as a co-infection or superinfection in 11–35% of cases, so the assumption was that a similar proportion of bacterial infections might be expected in COVID-19 as well [7]. However, studies showed that the coinfection rate during COVID-19 is rather low, yet antibiotics were still prescribed to most of these patients [6,8,9]. The second reason for antibiotic use to treat viral disease was the possibility of antiviral activity that some antibiotics showed in vitro. Azithromycin, a macrolide antibiotic has in vitro antiviral properties, such as decreased viral replication due to blocking the viral entrance into the host cells. Another in vitro study demonstrated synergistic activity of the combination of hydroxychloroquine and azithromycin against SARS-CoV-2, but other clinical studies did not confirm the benefit of this treatment, and because of the other possible side effects, like QT interval prolongation, this antibiotic is no longer recommended to be empirically used [10]. The third reason for the antibiotic treatment of COVID-19 is the usual differential diagnostic process. Most patients admitted to hospitals with COVID-19 are significantly sick, and sometimes it is extremely difficult to distinguish if the disease is of viral or bacterial origin. In day-to-day practice, the usual inflammatory markers are used to distinguish between the two etiologies, yet these parameters may not be effective in diagnosing COVID-19. Several studies showed that in COVID-19 without any bacterial infection, serum C-reactive protein levels can be very high, most probably as the result of intense immune response [11]. Similarly, even serum procalcitonin levels might be high during the possible cytokine storm syndrome commonly seen in COVID-19, although procalcitonin is considered a rather specific acute phase reactant during bacterial infections [12]. 

These practices have resulted in a significant overuse of antibiotics, so it is reasonable to expect that this has accelerated the process of bacterial resistance development. Almost all studies dealing with the impact of COVID-19 on bacterial resistance have confirmed the emergence of resistant bacteria causing health care-associated infections (HAIs) in COVID-19 patients [2]. Once resistant bacteria emerge, they will spread causing HAIs in other non-COVID-19 patients, health care workers, and even to the community. The aim of this study was to assess a hospital’s level of antibiotic consumption during the COVID-19 pre-pandemic and pandemic periods and to correlate these data with the rate of resistant microorganisms detected in all the patients hospitalized during the study period. 

## 2. Results

We gathered data on the number of patients admitted to the hospital and the consumption of observed antibiotics. More patients were admitted to the hospital during the pre-COVID-19 era, when compared to the COVID-19 period (132,828 versus 119,191 total admissions; 15,067 versus 16,602 median annual admissions; *p* = 0.021). At the same time, more patients during the COVID-19 period were treated with monitored antibiotics, especially CFT and VANC, when compared to the pre-COVID-19 phase. The consumption trends of PTZ (53 versus 56 median ACIs; *p* = 0.528) and MER (19 versus 16 median ACIs; *p* = 0.834) were not significantly different between the two periods. Contrary to this, the VANC (11 versus 7 median ACIs) and CFT (19 versus 16 median ACIs) consumptions were significantly higher during the COVID-19 period (compared to the pre-COVID-19 era), and these differences were statistically significant as the *p*-values were 0.001 and 0.036, respectively (Table 1).

The total number of MRSA, VRE, and *C. auris* isolates during the pre-COVID-19 and COVID-19 periods is presented in Table 2. During the pre-COVID-19 period, MRSA was detected more frequently compared to the COVID-19 period. While the difference for MDR-PA was only marginally significant, MRSA isolates were significantly less often detected during the COVID-19 period. There were no significant differences between the two study periods when analyzing the isolation of MDR-AB, CRE, and ESBL microorganisms. However, VRE, and *C. auris* were more often detected during the COVID-19 period with *p*-values of 0.026 and 0.004, respectively. The most evident increase in the VRE isolates was observed during the second half of the COVID-19 period (in 2021), but the number of *C. auris* isolates started to increase during the last quarter of 2020 when the COVID-19 pandemic had already reached Oman. 

Table 3 presents the distribution of isolated MDR microorganisms in different samples, but only those whose isolation rate was significantly different between the study periods. There were no statistically significant differences in the MDR isolation rates from blood cultures during the study period, but there was a difference when analyzing the isolates from urine cultures. CRE and VRE, as well as *C. auris*, were significantly detected more often from urine culture during the COVID-19 period with p-values of 0.011, 0.002, and 0.03, respectively. During the COVID-19 period, ESBL bacteria were more often detected in the endotracheal secretion culture (*p* = 0.018), while *C. auris* was more often isolated in the screening samples. At the same time, in screening samples MRSA, MDR-AB, and ESBL, bacteria were less frequently isolated during the COVID-19 period, and these differences were statistically significant with *p*-values of 0.004, 0.031, and 0.005, respectively.

Table 4 presents the correlations between each of analyzed antibiotic ACIs and the isolated microorganisms in the four-year study period. Increased use of PTZ was significantly positively correlated with increased isolation rate of ESBL microorganism (*rho* = 0.57; *p* = 0.021), while the increased isolation of VRE (*rho* = 0.58; *p* = 0.017) and *C. auris* (*rho* = 0.71; *p* = 0.002) was positively correlated with the increased use of VANC. However, contrary to scientific expectation, the increased use of VANC was negatively correlated with the microorganism MRSA (*rho* = −0.56; *p* = 0.025) and MDR-PA (*rho* = −0.61; *p* = 0.013). The increased use of CFT was positively correlated with the emergence of *C. auris* (*rho* = 0.56; *p* = 0.023).

## 3. Discussion

When comparing the pre-COVID-19 and COVID-19 periods, the results of this study show that the number of hospitalized patients during the pandemic period was significantly lower. During this same period, the proportion of patients treated with antibiotics was significantly higher. The consumption of PTZ was higher during the pre-COVID-19 period, but that was not statistically significant. On the other hand, the consumption of CFT and VANC was significantly higher during the COVID-19 period. 

Similar increased consumption of antibiotics was reported in other studies. Andrews and coauthors analyzed the antibiotic consumption in England during the period 2015–2020. The analysis included primary and secondary care. Prior to the COVID-19 pandemic, the total antibacterial consumption in England was steadily decreasing, but when the pandemic started, the antibiotic consumption steeply increased, particularly the consumption of broad-spectrum antibiotics prescribed for respiratory infections. In hospitals, the increase in consumption was predominantly due to consumption of antibiotics from the WHO “Watch” list [13]. In their review of antibiotic consumption during COVID-19 patients’ treatments, Malik and Mundra found that more than 78% of these patients were treated with antibiotics. The most frequently prescribed antibiotics were azithromycin, ceftriaxone, moxifloxacin, MER, and tazobactam. Additionally, there were also no significant differences in antibiotic consumptions while treating patients with severe or mild COVID-19 [14]. Although the WHO recommended avoiding antibiotic usage for mild and moderate COVID-19 cases, and when antibiotic treatment necessitates the use of antibiotics from the WHO “Access” list, the number of patients treated with broad-spectrum antibiotics increased significantly during the pandemic. This raises legitimate concerns about AMR. 

In our study, MRSA, MDR-PA, and MDR-AB were isolated less frequently during the COVID-19 period. While the differences were not significant for MDR-AB and MDR-PA, MRSA was isolated significantly less frequently. To a certain extent this, could be explained with the fact that during the COVID-19 period, health care workers were very compliant with donning personal protective equipment and hand hygiene rules. While ESBL, CRE, VRE, and *C. auris* were detected more often during the COVID-19 period, the increased isolation frequency for ESBL and CRE was not significant. When analyzing the isolation frequency timeline of VRE, it is evident that the isolation rate started to increase approximately six months after the COVID-19 pandemic started. This implies that the inappropriate antibiotic usage during the COVID-19 period potentially generated this resistance. 

Similar increases in isolation rates of resistant microorganisms have been reported in other studies. Gaspar and coauthors analyzed bacterial isolates from clinical and screening samples obtained from the patients treated in the ICU. They compared the isolated bacteria resistance pattern from the pre-COVID-19 and COVID-19 periods. During both periods, *K. pneumoniae* was isolated most often (53%), followed by *Acinetobacter baumannii* (37%), and *S. aureus* (10%). They found that during the COVID-19 period, the rate of resistance to carbapenems and polymyxin B significantly increased in both *K. pneumoniae* and *A. baumannii*; however, they did not report the resistance pattern for *S. aureus* [15]. In their retrospective study, Petrakis and coauthors compared the resistance pattern of bacteria isolated from blood and respiratory samples during pre-pandemic years (2018–2019) and through the pandemic period (2020–2022). They reported that the total number of isolated Gram-negative and Gram-positive bacteria from patients hospitalized in wards as well as in the ICU increased during the pandemic period. At the same time, the authors found that in isolated *A. baumannii*, *P. aeruginosa*, and *K. pneumoniae,* the resistance to carbapenems and colistin significantly increased during the pandemic period, regardless of if the patients were hospitalized in wards or ICUs. For Gram-positive bacteria, the authors reported that the resistance to vancomycin in *E. faecium* increased significantly, but also, they reported an increase in the MRSA isolation rate [16]. Hurtado and colleagues analyzed the antibiotic consumption and resistance pattern in six commonly isolated bacteria in 31 Colombian hospitals (*S. aureus*, *K. pneumoniae*, *E. coli*, *P. aeruginosa*, *A. baumannii* complex, and *E. faecium*). They compared the data from the pre-pandemic (March 2018 to July 2019) and pandemic period (March 2020 to July 2021). They also found that the total number of isolates increased during the pandemic period, and *K. pneumoniae* was the bacteria isolated most frequently during both analyzed periods. Their results showed that out of all isolated *S. aureus*, the proportion of MRSA remained the same, as well as the proportion of ESBL *E. coli*, as well as *E. coli* resistant to carbapenems; however, the proportion of ESBL and CRE *K. pneumoniae* significantly increased during the COVID-19 period. Likewise, a significant increase in carbapenem resistance was detected in *A. baumannii*, and *P. aeruginosa*. The study reported a significant increase in the isolation rate of VRE during the pandemic period. The authors also found that the consumption of all monitored antibiotics (CFT, cefepime, ciprofloxacin, PTZ, and VANC) significantly increased during the pandemic period. Only the consumption of MER in ICU settings slightly decreased [17].

In our study, the correlation analysis suggests that the overuse of broad-spectrum antibiotics resulted in a significant increase in AMR, and this effect was seen over a rather short time. Although the PTZ consumption during the study periods did not significantly change, as per the correlation analysis, this antibiotic led to the increased isolation frequency of ESBL producing bacteria. Most likely, this means that PTZ is permanently overused causing the AMR emergence. This is supported with a previous study which showed that PTZ was the most commonly prescribed antibiotic in the Royal Hospital [18]. In their study of more than 5.5 million hospitalized patients from 271 facilities in the United States, Bauer and colleagues found that out of all resistant bacteria only ESBL producers and VRE were more often isolated during the COVID-19 period. This AMR increase was driven by multiple factors, mainly antibiotic overuse, longer antibiotic treatment, use of inadequate empirical antibiotic, ICU admission, and length of stay in the hospital [19].

In the Royal Hospital, during the pre-COVID-19 period, VRE was rarely isolated, and it was not considered a resistant microorganism of great concern. Within the first few months of the COVID-19 pandemic, the number of VRE isolates significantly increased, and as our analysis showed, the increased VANC consumption potentially triggered this AMR. In their experiment, Baym and coauthors reported that the resistance in bacteria can be detected as early as 10–12 days, depending on the antibiotic to which the bacteria was exposed [20]. Most probably, the VANC overuse in our hospital generated the bacterial resistance followed by the VRE spread that was supported by all the other factors that help microbe spreading (e.g., inappropriate hand hygiene, lack of isolation precautions, and improper personal protective equipment use).

The results of our study show that the overuse of VANC and ceftriaxone is responsible for the increase in *C. auris* isolation. In their meta-analysis, Thomas-Rüddel and coauthors found that apart from widely recognized risk factors for invasive fungal infections (total parenteral nutrition, *Candida* colonization, (abdominal) surgery, broad-spectrum antibiotics, sepsis); renal replacement therapy, mechanical ventilation, blood transfusion, and diabetes are additional important risk factors [21]. In our hospital, the first case of a COVID-19 patient was admitted in February 2020 [22], but at the beginning of the pandemic, the burden of COVID-19 cases was not heavy, and there were no significant changes in the antibiotic consumption. The VANC and CFT consumption started to rise steadily during the COVID-19 period, and at the same time, the *C. auris* isolation rate also started to increase, suggesting that antibiotic overuse was at least one of the factors that triggered the *C. auris* emergence. 

Our study has a few limitations as the analyzed data originated from only one center, which can bias the conclusions. Although the Royal Hospital is the biggest tertiary care facility in Oman, where patients are referred from the whole country, the local bacterial resistance pattern might not represent the situation in other hospitals. Another limitation is that the consumption of only four antibiotics was analyzed, and the consumption of other antibiotics might also affect the AMR. 

## 4. Materials and Methods

This retrospective study was conducted at the Royal Hospital, Muscat, Oman, after the approval of the hospital’s Scientific Research Committee (SRC#95/2021). The RH is the main tertiary hospital in Oman, and it was designated nationally for the care of COVID-19 patients [8]. It has 1000 beds and approximately 60,000 hospital admissions annually. When the COVID-19 pandemic started, all elective admissions and procedures were postponed or the patients were referred to other healthcare facilities. The four years of data were extracted from the Royal Hospital database. As per the health care system in Oman, patients > 13 years of age are treated in adult wards, so all the patients hospitalized in these wards were enrolled in the study. The data were collected from all adult wards and intensive care units (ICUs). The years 2018 to 2019 represented the pre-COVID-19 phase, while the years 2020 to 2021 represent the COVID-19 period. 

### 4.1. Antibiotic Consumption Analysis

In the Royal Hospital, the most commonly used antibiotics for empirical treatment are ceftriaxone (CFT), piperacillin-tazobactam (PTZ), meropenem (MER), and vancomycin (VANC) [8]. These antibiotics are included in the World Health Organization (WHO) “Watch” list as they have higher resistance potential and should be prioritized as key targets of stewardship programs and monitoring [23]. The consumption of these four antibiotics was analyzed using the WHO anatomical therapeutic chemical classification/defined daily dose (ATC/DDD) index guidelines [24]. As this system does not take into consideration possible different drug doses in different patients, it only gives a rough estimation of drug consumption. The three months of consumption for each of the analyzed antibiotics was presented in grams and used to calculate the DDD. The consumption rate was presented as the antibiotic consumption index (ACI), which is the ratio of DDD per 100 bed days. 

### 4.2. Microorganism Analysis

Clinical samples used to detect resistant microorganisms were blood cultures (b/c), urine cultures (u/c), and endotracheal secretion cultures (ET/c) taken during the hospitalization of every enrolled patient. Screening swab culture results (s/c) were also used to analyze the rate of resistant microorganisms carriers. Screening for multidrug-resistant organisms (MDRO) in the Royal Hospital is routinely performed following the hospital MDRO Screening Policy [25].

### 4.3. Surveillance of MDRO per Royal Hospital Policy

MDR-*Acinetobacter* spp. (MDR-AB): any *Acinetobacter* spp. testing non-susceptible (i.e., resistant or intermediate) to at least one agent in at least three out of six antimicrobial classes (ampicillin/sulbactam; cephalosporins; b-lactam/b-lactamase inhibitor combination; carbapenems; fluoroquinolones; aminoglycosides).

Carbapenem-resistant Enterobacterales (CRE): any *Escherichia coli*, *Klebsiella oxytoca*, *Klebsiella pneumoniae*, or *Enterobacter* spp. resistant to imipenem, MER, doripenem, or ertapenem.

Methicillin-resistant *Staphylococcus aureus* (MRSA): *S. aureus* testing resistant to oxacillin, cefoxitin, or methicillin. 

Vancomycin-resistant *Enterococcus* spp. (VRE): *Enterococcus faecalis*, *Enterococcus faecium*, or *Enterococcus* spp. unspecified if resistant to vancomycin. 

MDR-*Pseudomonas aeruginosa* (MDR-PA): any *Pseudomonas* spp. testing non-susceptible (i.e., resistant or intermediate) to at least one agent in at least three out of five antimicrobial classes (cephalosporins; b-lactam/b-lactamase inhibitor combination; carbapenems; fluoroquinolones; aminoglycosides). 

Extended-spectrum b-lactamase enzyme (ESBL) producer: bacteria that produce enzymes that mediate resistance to third-generation cephalosporins, monobactams, but not on cephamycins (e.g., cefoxitin, cefotetan) or carbapenems (e.g., imipenem, MER). 

*Candida auris*: yeast that has elevated minimum inhibitory concentration to one or more of the three major anti-fungal classes: azoles (e.g., fluconazole); amphotericin B; echinocandins (e.g., caspofungin). 

### 4.4. Statistical Analysis

Medians and interquartile ranges were used to present the data, while analyses were performed using the Mann–Whitney U test for trend across the study period (2018–2021). The correlation between each of the antibiotic ACIs and the isolated microorganisms in the four-year study period was performed using Spearman’s rank correlation coefficient. A two-tailed level of significance was set at *p* < 0.05 level. Statistical analyses were conducted using STATA version 16.1 (STATA Corporation, College Station, TX, USA).

## 5. Conclusions

This study confirms that the overuse of antibiotics triggers the development of bacterial resistance. The emergence of resistant bacterial species depends on the previous local AMR situation, but also on the type of antibiotics that are used, overused, and/or misused. The results of this study emphasize the importance of urgent, strict, and permanent antibiotic control through adequate antimicrobial stewardship activities, as the development of bacterial resistance is fast, yet the process of decreasing the number of resistant bacteria is slow. Future comprehensive research projects should try to correlate the AMR and different antimicrobial stewardship interventions in order to define the most effective stewardship approaches in controlling the emergence of bacterial resistance. 

## Figures and Tables

**Table 1:** Distribution of admitted patients and the antibiotic consumption.

Period Quarter/Year	Admission	Pt on ABX	%	PTZ DDD	PTZ ACI	MER DDD	MER ACI	VANC DDD	VANC ACI	CFT DDD	CFT ACI
I/2018	16,668	916	5.5	3883	38.8	1580	15.8	655	6.6	2353	23.5
II/2018	15,501	809	5.2	4365	43.7	3185	31.9	638	6.4	1928	19.3
III/2018	15,987	864	5.4	5626	56.3	1610	16.1	642	6.4	1985	19.9
IV/2018	17,360	980	5.6	6275	62.8	1699	17	718	7.2	2148	21.5
I/2019	16,955	871	5.1	3884	38.8	1154	11.5	601	6	1804	18
II/2019	16,192	917	5.7	5540	55.4	2812	28.1	669	6.7	1704	17
III/2019	16,537	945	5.7	5931	59.3	1399	14	624	6.2	1948	19.5
IV2019	17,628	1048	5.9	7231	72.3	1519	15.2	729	7.3	2399	24
Total pre-COVID-19	132,828	7350	5.53	42,735		14,958		5276		16,269	
Median (IQR) ACI				56 (41–61)		16 (14–23)		7 (6–10)		20 (19–23)	
I/2020	16,362	1204	7.4	5573	55.7	1278	12.8	799	8	2532	25.3
II/2020	11,743	831	7.1	3777	37.8	1443	14.4	739	7.4	2407	24.1
III/2020	13,665	891	6.5	4947	49.5	2142	21.4	1333	13.3	1882	18.8
IV/2020	14,933	886	5.9	4356	43.6	1894	18.9	1116	11.2	2381	23.8
I/2021	16,001	894	5.6	5588	55.9	1393	13.9	879	8.8	1944	19.4
II/2021	14,545	1064	7.3	5356	53.6	2176	21.8	1271	12.7	3160	31.6
III/2021	15,201	1044	6.9	6865	68.7	1986	19.9	1128	11.3	2446	24.5
IV/2021	16,741	1101	6.6	5283	52.8	1814	18.1	980	9.8	2488	24.9
Total COVID-19	119,191	7915	6.64	41,745		14,126		8245		19,240	
Median (IQR) ACI				53 (47–56)		19 (14–21)		11 (8–12)		24 (22–25)	
*p*-value				0.528		0.834		0.001		0.036	

Legend: Pt on ABX = Number of patients on antibiotic treatment; PTZ = piperacillin tazobactam; MER = meropenem; VANC = vancomycin; CFT = ceftriaxone; DDD = defined daily doses; ACI = antibiotic consumption index; IQR = interquartile range.

**Table 2:** Comparison of total number of isolated MRSA, VRE, and *C. auris* during the study periods.

Period	Pre-COVID-19									COVID-19									*p*-Value
Year	2019				2020				Median	2021				2022				Median	
Quarter	I	II	III	IV	I	II	III	IV		I	II	III	IV	I	II	III	IV		
MRSA	174	150	173	170	170	154	176	162	170	143	115	117	143	130	147	153	178	143	0.016
VRE	4	4	1	3	4	2	3	1	3	2	2	10	7	35	86	227	184	23	0.026
*C. auris*	0	0	0	2	0	0	3	20	0	36	14	17	28	23	19	10	17	18	0.004

Legend: MRSA = methicillin-resistant *Staphylococcus aureus*; VRE = vancomycin-resistant *Enterococci*; *C. auris* = *Candida auris.*

**Table 3:** Distribution of MDR isolates from specific samples during the study periods.

Period	Pre-COVID-19									COVID-19									*p*-Value
Year	2019				2020				Median	2021				2022				Median	
Quarter	I	II	III	IV	I	II	III	IV		I	II	III	IV	I	II	III	IV		
Sample	Urine culture																		
CRE	16	16	10	26	16	18	10	16	16	29	11	22	24	28	31	29	27	28	0.011
VRE	0	0	0	1	0	1	2	0	0	2	1	5	3	8	10	38	22	6.5	0.002
*C. auris*	0	0	0	0	0	0	0	1	0	0	0	3	6	4	3	1	0	2	0.03
Sample	Endotracheal secretion culture																		
ESBL	9	10	11	6	9	15	5	11	10	7	10	31	39	18	30	32	16	24	0.018
Sample	Screening																		
MRSA	163	144	153	157	158	147	162	143	155	128	102	98	122	107	124	131	157	123	0.004
MDRAB	63	58	64	56	52	41	41	26	54	33	15	28	26	17	44	47	42	31	0.031
ESBL	3	2	2	2	2	4	3	0	2	0	0	0	2	0	0	0	1	0	0.005
*C. auris*	0	0	0	1	0	0	1	15	0	33	14	14	21	18	16	9	16	16	0.002

Legend: MRSA = methicillin-resistant *S. aureus*; CRE = carbapenem-resistant *Enterobacterales*; VRE = vancomycin-resistant *Enterococci*; MDR AB = multidrug-resistant *A. baumannii*; ESBL = extended-spectrum beta-lactamase; *C. auris* = *Candida auris*.

**Table 4:** Correlation between the antibiotic ACI and the MDR isolates during the study period.

	Piperacillin Tazobactam		Meropenem		Vancomycin		Ceftriaxone	
Microorganism	*rho*	*p*-Value	*rho*	*p*-Value	*rho*	*p*-Value	*rho*	*p*-Value
MRSA	0.25	0.351	−0.1	0.716	−0.56	0.025	−0.05	0.85
CRE	0.06	0.816	−0.38	0.144	0.39	0.14	0.36	0.165
VRE	−0.16	0.565	0.32	0.229	0.58	0.017	0.18	0.495
MDR PA	−0.12	0.665	−0.35	0.182	−0.61	0.013	−0.27	0.321
MDR AB	−0.11	0.684	0.4	0.124	0.19	0.484	0.05	0.867
ESBL	0.57	0.021	0.15	0.579	0.46	0.075	0.42	0.102
*C. auris*	0.17	0.539	−0.2	0.468	0.71	0.002	0.56	0.023

Legend: ACI = antibiotic consumption index; MDR = multidrug resistant; MRSA = methicillin-resistant *S. aureus*; CRE = carbapenem-resistant *Enterobacterales*; VRE = vancomycin-resistant *Enterococci*; MDR PA = multidrug-resistant *P. aeruginosa*; MDR AB = multidrug-resistant *A. baumannii*; ESBL = extended-spectrum beta-lactamase; *C. auris* = *Candida auris*.

## Data Availability

The data presented in this study are available on request from the corresponding author. The data are not publicly available due to the Royal Hospital policy.
